# Mortality prediction of heart transplantation using machine learning models: a systematic review and meta-analysis

**DOI:** 10.3389/frai.2025.1551959

**Published:** 2025-04-04

**Authors:** Ida Mohammadi, Setayesh Farahani, Asal Karimi, Saina Jahanian, Shahryar Rajai Firouzabadi, Mohammadreza Alinejadfard, Alireza Fatemi, Bardia Hajikarimloo, Mohammadhosein Akhlaghpasand

**Affiliations:** ^1^Student Research Committee, School of Medicine, Shahid Beheshti University of Medical Sciences, Tehran, Iran; ^2^Cardiovascular Surgery Research and Development Committee, Iran University of Medical Sciences (IUMS), Tehran, Iran

**Keywords:** heart transplant, machine learning, mortality, artificial intelligence, heart failure

## Abstract

**Introduction:**

Machine learning (ML) models have been increasingly applied to predict post-heart transplantation (HT) mortality, aiming to improve decision-making and optimize outcomes. This systematic review and meta-analysis evaluates the performance of ML algorithms in predicting mortality and explores factors contributing to model accuracy.

**Method:**

A systematic search of PubMed, Scopus, Web of Science, and Embase identified relevant studies, with 17 studies included in the review and 12 in the meta-analysis. The algorithms assessed included random forests, CatBoost, neural networks, and others. Model performance was evaluated using pooled area under the curve (AUC) values, with subgroup analyses for algorithm type, validation methods, and prediction timeframes. The risk of bias was assessed using the QUADAS-2 tool.

**Results:**

The pooled AUC of all ML algorithms was 0.65 (95% CI: 0.64, 0.67), with no significant difference between machine learning and deep learning models (*p* = 0.67). Among the algorithms, CatBoost demonstrated the highest accuracy (AUC 0.80, 95% CI: 0.74, 0.86), while K-nearest neighbor had the lowest accuracy (AUC 0.53, 95% CI: 0.50, 0.55). A meta-regression indicated improved model performance with longer post-transplant periods (*p* = 0.008). When pooling only the best-performing models, the AUC improved to 0.73 (95% CI: 0.68, 0.78). The risk of bias was high in eight studies, with the flow and timing domains most commonly contributing to bias.

**Conclusion:**

ML models demonstrate moderate accuracy in predicting post-HT mortality, with CatBoost achieving the best performance. While ML shows potential for improving predictive precision, significant heterogeneity and biases highlight the need for standardized methods and further external validations to enhance clinical applicability.

**Systematic review registration:**

https://www.crd.york.ac.uk/PROSPERO/view/CRD42024509630, CRD42024509630

## Graphical abstract

Summary of the flow and findings of the paper. RF: random forest; XGB: extreme gradient boosting (XGBoost); ANN: artificial neural network; SVM: support vector machine; DT: decision tree; AUC: area under the curve.
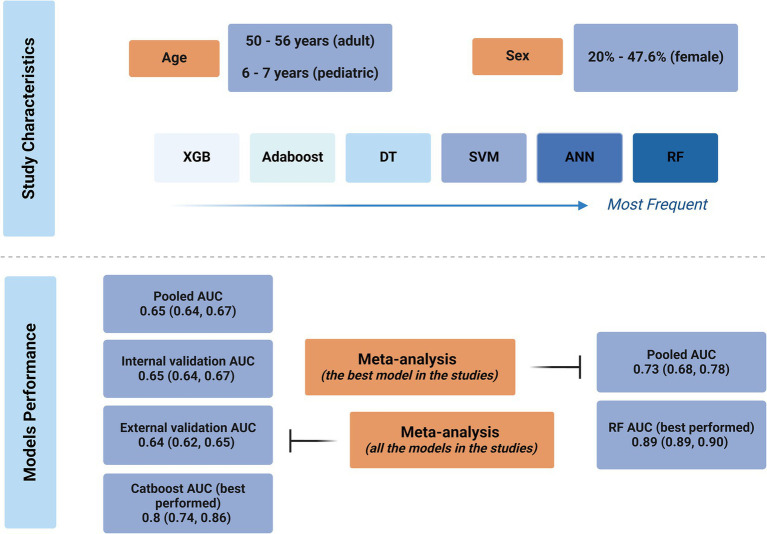

## 1 Introduction

Heart transplantation (HT) is a life-saving treatment for patients in the last stages of heart failure, providing them with a last chance for survival while also improving their quality of life (Awad et al., 2022). It remains, however, one of the most challenging procedures in medicine due to the very limited availability of a suitable donor heart, the intricacies in matching between the donor and the recipient, and the significant risks following transplantation, including graft rejection and infection (Vaidya et al., 2023; Khush et al., 2019). Accurate assessment and decision on eligibility, optimum donor matching, and close postoperative monitoring to prevent graft rejection are required at every step in HT. These challenges are compounded because physiological and immune variables are very complex and vary greatly between individuals, thus placing an increased demand for very accurate predictive tools to guide clinicians at every step.

Several risk-scoring models have been developed to help overcome some of these challenges and guide clinicians with regard to transplant viability and outcomes. Commonly used ones include the Donor Risk Index (DRI) (Weiss et al., 2012), the risk stratification score (RSS) (Hong et al., 2011), and the Index for Mortality Prediction After Cardiac Transplantation (IMPACT) (Weiss et al., 2011). These regression-based models depend on different clinical and donor-related variables to estimate patient risk and predict mortality after transplantation. While these models are helpful, there is an inherent limitation in the specificity and generalizability of many regression-based models toward complicated and personalized transplant issues.

Only recently, artificial intelligence (AI) and machine learning (ML) have emerged as strong alternatives to the conventional risk-scoring model, offering higher predictive accuracy and customization (Maleki Varnosfaderani and Forouzanfar, 2024). While conventional models rely on pre-specified variables and linear relationships, AI and ML algorithms analyze vast volumes of diverse and complex data—identifying patterns and interactions that would have gone undetected with traditional approaches. Advanced methods include neural networks, ensemble methods, and deep learning, which further empower AI-driven predictive models to make more personalized predictions regarding real-time physiological changes, thus allowing dynamic decision-making across the transplant continuum (Ravindhran et al., 2023). These represent some of the key opportunities regarding risk stratification, donor–recipient matching, and post-transplant monitoring, all of which adapt to the unique profile of each patient in a manner that may potentially reduce post-transplant mortality and improve long-term outcomes (Guijo-Rubio et al., 2020).

This systematic review and meta-analysis aimed to assess the performance of ML models for HT by focusing on AUC as an indication of predictive accuracy. The review aimed to underline the clinical potential of AI-based models in predicting transplant outcomes and contribute to the growing body of evidence that supports the use of AI in HT.

## 2 Materials and methods

This systematic review and meta-analysis followed the Preferred Reporting Items for Systematic Reviews and Meta-Analyses (PRISMA) guidelines to identify studies that develop or validate artificial intelligence methods for predicting HT mortality. The research protocol has been registered on PROSPERO and is accessible at CRD42024509630.

### 2.1 Search strategy

A comprehensive systematic review was conducted on 12 May 2024, utilizing four bibliographic databases: PubMed, Embase, Scopus, and Web of Science. The search was conducted with MeSH terms and their synonyms for “heart transplant,” “Artificial Intelligence,” and “mortality.” The search terms were appropriately adjusted for each specific database. There were no limitations specified regarding the year of publication. To ensure a thorough and unbiased selection process, two authors independently assessed the retrieved articles to determine their inclusion. A third reviewer solved possible inter-reviewer discrepancies and disagreements between the two initial reviewers.

### 2.2 Eligibility criteria

The eligibility criteria comprised the following aspects: Inclusion was limited to studies using well-established research designs, including prospective and retrospective cohort studies, case–control and experimental studies, and randomized controlled trials (RCTs). Narrative reviews, meta-analyses, case reports, animal studies, conference abstracts, editorials, and commentaries were excluded. In addition, studies that were not written in English were excluded.

Studies were included based on the following Population, Intervention, Comparison, and Outcome (PICO) criteria:

Population: The population of interest consisted of patients undergoing cardiac transplantation.

Intervention: The interventions assessed were predictive models utilizing artificial intelligence. These models could either be in the development stage or undergoing validation. For this study, we excluded studies that used linear regression (LR) models exclusively.

Comparator: When applicable, comparisons were made with standard clinical care practices or non-AI predictive models.

Outcomes: The primary outcome of interest was the area under the receiver operating characteristic (AUC) for mortality prediction. This metric was used to evaluate the performance of AI models, whether they were being developed or validated.

### 2.3 Study selection

Two authors independently selected articles based on established criteria through a two-phase process following a preliminary review of titles and abstracts. The impartial third-party reviewer resolved the conflicting viewpoints of the two authors. Subsequently, eligible studies underwent a comprehensive full-text evaluation.

### 2.4 Data extraction

Two authors independently conducted data extraction, and a third author made the final decision in case of any possible discrepancies. The data were extracted into a pre-constructed Excel sheet and included the first author, year of publication, country, population type (pediatric vs. adult) and size, population age and gender, post-transplant mortality timeframe, algorithms used, the best performing algorithm, AUC and standard error or 95% confidence interval (95% CI), mode of validation, and type of validation (internal or external).

### 2.5 Quality assessment

The quality assessment of the included studies was performed using the QUADAS-2 tool to assess the risk of bias (Whiting et al., 2011). Each study was evaluated across four domains: patient selection, index test, reference standard, and flow and timing. Studies were classified as high risk, low risk, and unclear risk of bias.

### 2.6 Statistical analysis

The meta-analysis of the AUC of the included studies was conducted using Stata version 18 (StataCorp. 2023, Stata Statistical Software, College Station, TX, United States). A random-effects model was used due to heterogeneous machine learning algorithms. Internally and externally validated models were separated for the main meta-analysis. Heterogeneity was evaluated using the I^2^ statistic, with values greater than 50% indicating substantial heterogeneity. To investigate heterogeneity, sub-group analysis by the type of algorithm, machine learning or deep learning algorithms, and meta-regression by the time of mortality being predicted (i.e., 12 months, 3 months, and 120 months) was conducted. Subgroup differences in the subgroup analysis were determined using Pearson’s chi-squared test. Statistical significance was determined with a *p*-value threshold of less than 0.05. Sensitivity analysis was performed using the leave-one-out method and via the exclusion of studies with a high risk of bias. Publication bias was assessed using Egger’s regression test (with a *p*-value threshold of less than 0.05) if the meta-analysis included at least 10 studies.

## 3 Results

### 3.1 Study selection

**Figure 1:**
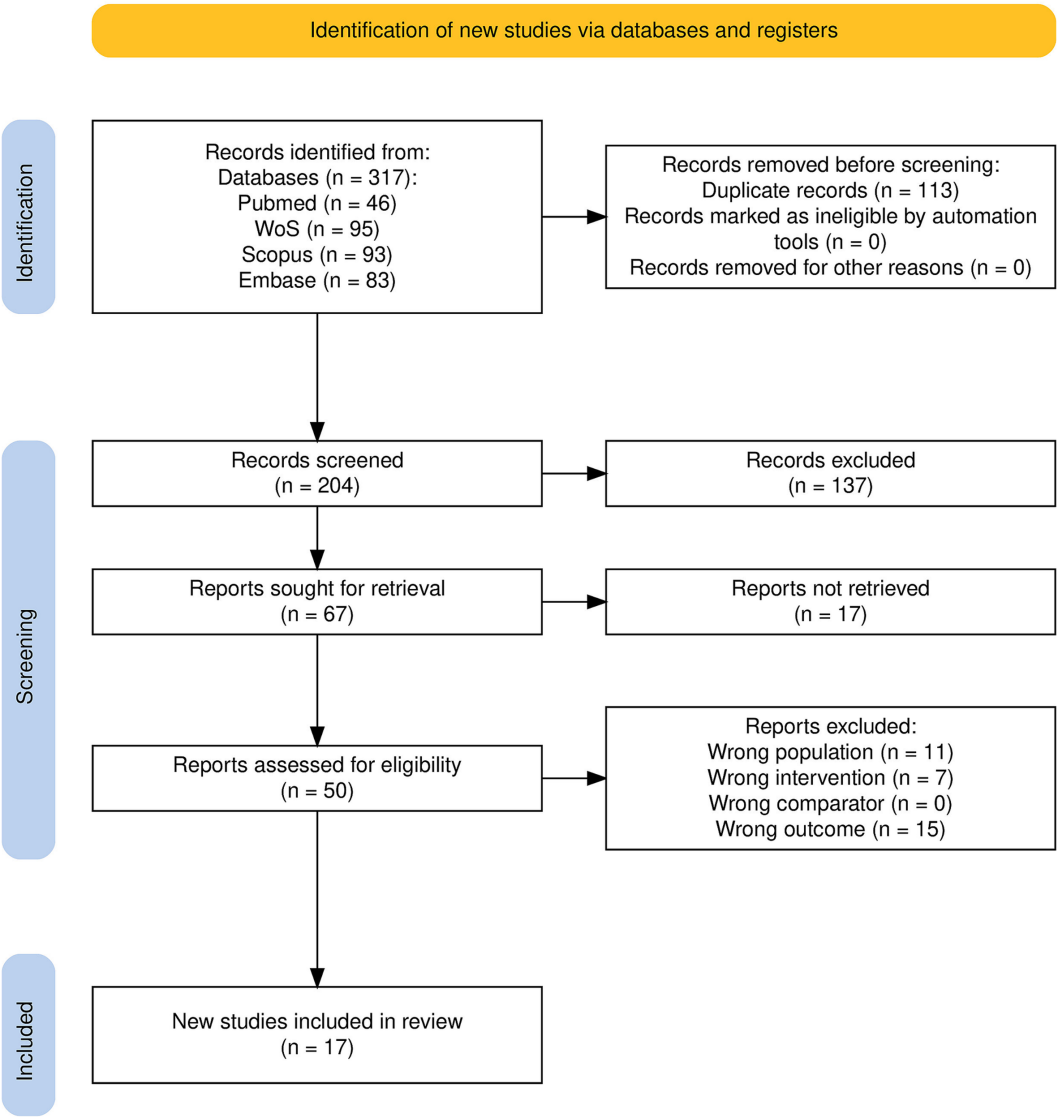
PRISMA flowchart.

Of the 317 articles identified during the initial search process, 204 remained after duplicate removal; 66 of these were selected for full-text retrieval and evaluation after title–abstract screening, and 17 records met the predefined inclusion criteria to be considered for the current systematic review. From these, a further 12 publications contained adequate data to be included in the meta-analysis (Figure 1).

### 3.2 Study characteristics

Detailed characteristic information is provided in Table 1. The included studies were published between 2015 and 2023, with 11 published since 2020. Most of the studies (*n* = 13) utilized an adult population (Nilsson et al., 2015; Dag et al., 2017; Medved et al., 2018; Yoon et al., 2018; Miller P. E. et al., 2019; Agasthi et al., 2020; Zhou et al., 2021; Kampaktsis et al., 2023; Kampaktsis et al., 2021; Ayers et al., 2021; Raji and Safna, 2022; Lisboa et al., 2022; Shou et al., 2022), three studies used a pediatric population (Miller R. et al., 2019; Killian et al., 2023; Ashfaq et al., 2023), and one study pooled adults and children in their sample population (Miller et al., 2022). The populations were mostly sampled from the American United Network for Organ Sharing (UNOS) registry (*n* = 14) (Dag et al., 2017; Medved et al., 2018; Yoon et al., 2018; Miller P. E. et al., 2019; Kampaktsis et al., 2023; Kampaktsis et al., 2021; Ayers et al., 2021; Raji and Safna, 2022; Lisboa et al., 2022; Shou et al., 2022; Miller R. et al., 2019; Killian et al., 2023; Ashfaq et al., 2023; Miller et al., 2022), while the International Society for Heart and Lung Transplantation (ISHLT) registry was used by two studies (Nilsson et al., 2015; Agasthi et al., 2020), the Scientific Registry of Transplant Recipients (SRTR) by one study (Lisboa et al., 2022), the Nordic Thoracic Transplantation Database by one study (Nilsson et al., 2015), and local medical records were used by one study (Zhou et al., 2021). Population sizes ranged from 381 (Zhou et al., 2021) to 67,939 (Miller et al., 2022) participants. The mean or median ages of the participants were mostly between 50 and 56 years old for the adult populations and between 6 and 7 years old for the pediatric populations. The ratio of females in the included studies ranged between 20% (Nilsson et al., 2015) and 47.6% (Raji and Safna, 2022), yet for six studies, the percentage of female participants was unspecified (Dag et al., 2017; Yoon et al., 2018; Miller P. E. et al., 2019; Agasthi et al., 2020; Lisboa et al., 2022; Miller R. et al., 2019). Regarding post-transplant mortality, most studies investigated 1-year mortality (*n* = 15), yet the time points ranged from 3 months (Yoon et al., 2018; Miller et al., 2022) to 10 years (Yoon et al., 2018).

**Table 1:** Characteristics of the included studies.

Author, year of publication, country	Population (adult or pediatric)	Data source	Population size	Mean age ± SD	Gender (female %)	Outcomes	Algorithms; best performing model (AUC)	Mode of validation	Type of validation
Nilsson et al. (2015), Sweden	Adult	ISHLT registry + Nordic Thoracic Transplantation Database	56,625 transplants	Train: median 54 Internal validation: median 54 External validation: median 52	Train: 20% Internal validation: 22.3% External validation: 22%	1-year mortality	ANN; ANN (0.64)	5-fold cross validation	Internal validation + external validation
Dag et al. (2017), USA	Adult	UNOS registry	15,580	NR	NR	1-, 5-, and 9-year mortality	SVM, ANN, DT; SVM (0.83)	10-fold cross validation	Internal validation
Medved et al., 2018, UK	Adult	UNOS registry	27,705 patients (train: 22,263; IV:5,597)	52 ± 13	24%	1-year mortality	ANN; ANN (0.65)	5-fold cross validation	External validation
Yoon et al. (2018), UK	Adult	UNOS registry	51,971	NR	NR	3 month, 1 year, and 3 year, and 10 year mortality	Trees of predictors, linear perceptron, Adaboost, Deepboost, Logitboost, XGB, DT, RF, NN; Trees of predictors (0.66)	5-fold cross validation	Internal validation
Miller P. E. et al. (2019) and Miller R. et al. (2019), USA	Pediatric	UNOS registry	Train: 1-year 2,545, 3-year 1,856, 5-year 1,285 Test: 1-year 635, 3-year 459, 5-year 320	NR	NR	1 year, 3 year, and 5 year mortality	RF, ANN; RF (0.72)	Train/ validation (80%/20%)	Internal validation
Miller P. E. et al. (2019) and Miller R. et al. (2019), India & USA	Adult	UNOS registry	56,477	NR	NR	1-year survival	NN, DT, SVM, RF, naïve-bayes; NN (0.66)	Train/ validation with bootstrapping	Internal validation
Agasthi et al. (2020), USA	Adult	ISHLT registry	15,236	NR	NR	5-year mortality	GBM; GBM (0.72)	10-fold cross-validation	Internal validation
Kampaktsis et al. (2021), USA	Adult	UNOS registry	18,625	53 ± 13	27%	1-year mortality	Adaboost, DT, SVM, KNN; Adaboost (0.69)	Train/ validation (75%/25%)	Internal validation
Ayers et al. (2021), USA	Adult	UNOS registry	33,657	52.8 ± 12.4	25%	1-year mortality	DNN, Adaboost, RF, Ensemble (DNN + Adaboost+RF+ logistic regression); Ensemble (0.76)	Train/ validation (80%/20%)	Internal validation
Zhou et al. (2021), China	Adult	Union Hospital, Tongji Medical College data	381	43.78 ± 16.45	23.8%	1-year mortality	SVM, RF, XGB, AdaBoost, GBM, ANN; RF (0.80)	Train/ validation with bootstrapping	Internal validation
Kampaktsis et al. (2023), USA	Adult	UNOS registry	1,033 patients	Median 34	38.9%	1-year and 3-year mortality	CatBoost; CatBoost (0.80)	Train/ validation (75%/25%)	Internal validation
Miller et al. (2022), USA	Adult/pediatric	UNOS registry	67,939 (59,590 adult+8,349 pediatric)	Median 54 (adult 55, pediatric 7)	27.4% (Adult: 25% pediatrics: 44.2%)	1-year and 90 days mortality	RF, XGB; RF (0.89)	10-fold cross-validation or rolling cross validation	Internal validation
Raji and Safna (2022), India	Adult	UNOS registry	485	51.19 ± 11.03	47.6%	survival prediction	MLP, ANN; ANN (0.95)	10-fold cross-validation	External validation
Lisboa et al. (2022), UK	Adult	UNOS, SRTR	42,185	Mean 52.3	24.2%	1-year mortality	Partial neural network, GBM, ANN; ANN (0.64)	Test/ validation	Internal and external validation
Shou et al. (2022), USA	Adult	UNOS registry	1,584	56	26.2%	1-year mortality	XGB; XGB (0.71)	Train/ validation (70%/30%)	Internal validation
Killian et al. (2023), USA	Pediatric	UNOS registry	8,201	Mean 6.78 ± 6.47	43.62%	1-, 3-, and 5-years mortality	XGB, SVM, RF, SGD, MLP, AdaBoost, NN; RF (0.76)	10-fold cross validation	Internal validation
Ashfaq et al. (2023), USA	Pediatric	UNOS registry	4,150	Mean 6.46	44.14%	1-year and 3-year survival	GBM, SVM, RF, DT; RF (0.68)	Train/ validation (70%/30%)	Internal validation

AUC: Area Under Receiver Operating Characteristics Curve, ISHLT: International Society for Heart and Lung Transplantation, UNOS: United Network for Organ Sharing, SRTR: Scientific Registry of Transplant Recipients, ANN: Artificial Neural Network, SVM: Support Vector Machine, DT: Decision Trees, RF: Random Forest, NN: Neural Network, GBM: Gradient Boosting Machine, KNN: K-Nearest Neighbor, DNN: Deep Neural Network, XGB: Extreme Gradient Boosting, SGD: Stochastic Gradient Descent, and MLP: Multi-Layer Perceptron.

The most used algorithms were random forest (RF) (Yoon et al., 2018; Miller P. E. et al., 2019; Zhou et al., 2021; Ayers et al., 2021; Miller R. et al., 2019; Killian et al., 2023; Ashfaq et al., 2023; Miller et al., 2022), artificial neural network (ANN) (Nilsson et al., 2015; Dag et al., 2017; Medved et al., 2018; Zhou et al., 2021; Raji and Safna, 2022; Lisboa et al., 2022; Miller R. et al., 2019), support vector machine (SVM) (Dag et al., 2017; Miller P. E. et al., 2019; Zhou et al., 2021; Kampaktsis et al., 2021; Killian et al., 2023; Ashfaq et al., 2023), decision tree (DT) (Dag et al., 2017; Yoon et al., 2018; Miller P. E. et al., 2019; Kampaktsis et al., 2021; Ashfaq et al., 2023), adaptive boosting (AdaBoost) (Yoon et al., 2018; Zhou et al., 2021; Kampaktsis et al., 2021; Ayers et al., 2021; Killian et al., 2023), extreme gradient boosting (XGB) (Yoon et al., 2018; Zhou et al., 2021; Shou et al., 2022; Killian et al., 2023; Miller et al., 2022), gradient boosting machine (GBM) (Agasthi et al., 2020; Zhou et al., 2021; Lisboa et al., 2022; Ashfaq et al., 2023), and neural network (NN) (Yoon et al., 2018; Miller P. E. et al., 2019; Killian et al., 2023), in descending order. Other used algorithms included multi-layer perceptrons (MLP) (Raji and Safna, 2022; Killian et al., 2023), K-nearest neighbor (KNN) (Kampaktsis et al., 2021), deep neural networks (DNNs) (Ayers et al., 2021), categorical boosting (CatBoost) (Kampaktsis et al., 2023), partial neural networks (Lisboa et al., 2022), stochastic gradient descent (SGD) (Killian et al., 2023), linear perceptrons (Yoon et al., 2018), deep boosting (DeepBoost) (Yoon et al., 2018), naïve Bayesian (Miller P. E. et al., 2019), logistic boosting (LogitBoost) (Yoon et al., 2018), trees of predictors (Yoon et al., 2018), and an ensemble model of DNN + AdaBoost + RF + LR (Ayers et al., 2021). Modes of validation were commonly train/validation splits (*n* = 9), followed by K-fold cross-validations (*n* = 8). External validation was only performed in four studies (Nilsson et al., 2015; Medved et al., 2018; Raji and Safna, 2022; Lisboa et al., 2022).

### 3.3 Performance of the models

**Figure 2:**
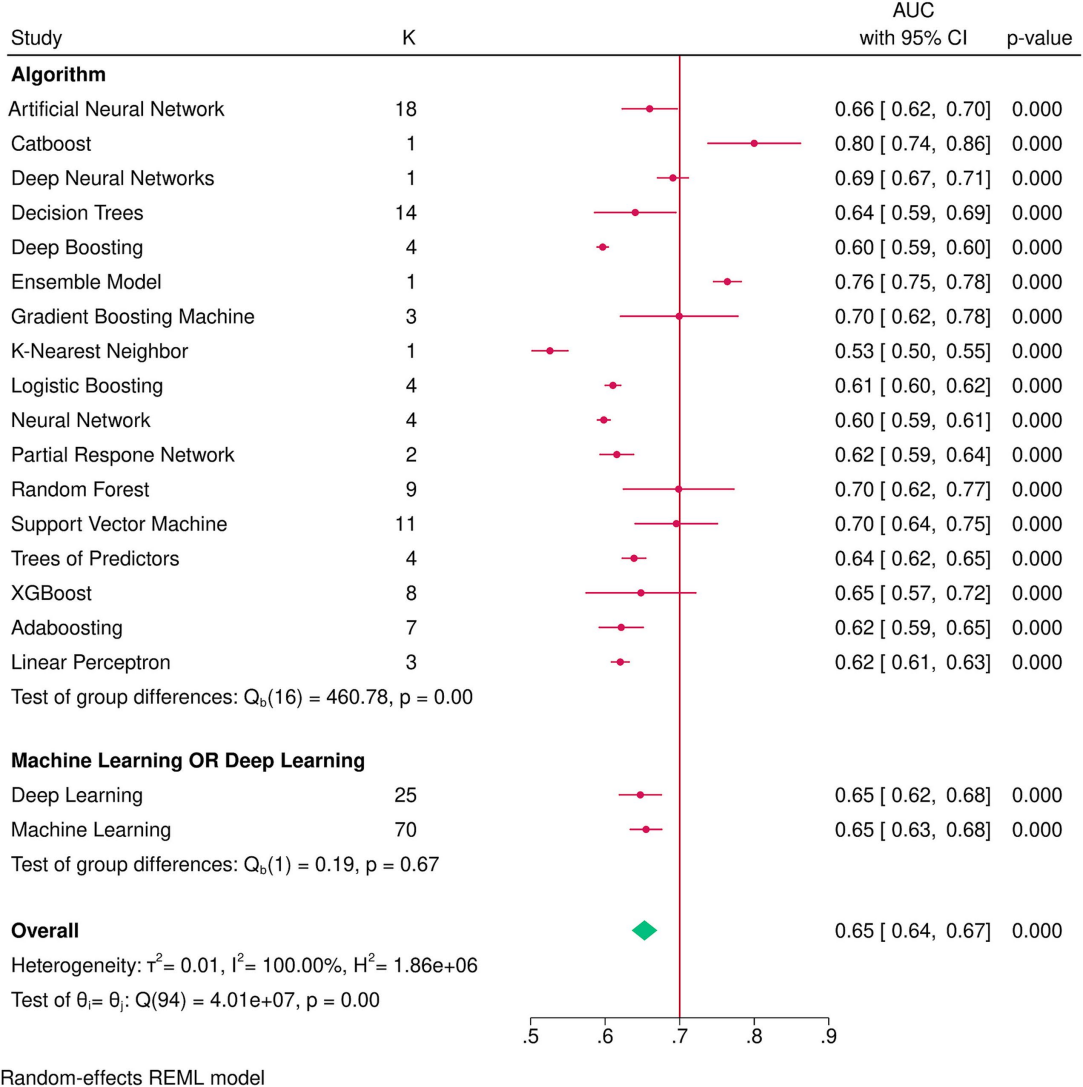
Subgroup analysis by the type of algorithm and machine learning vs. deep learning algorithms.

**Figure 3:**
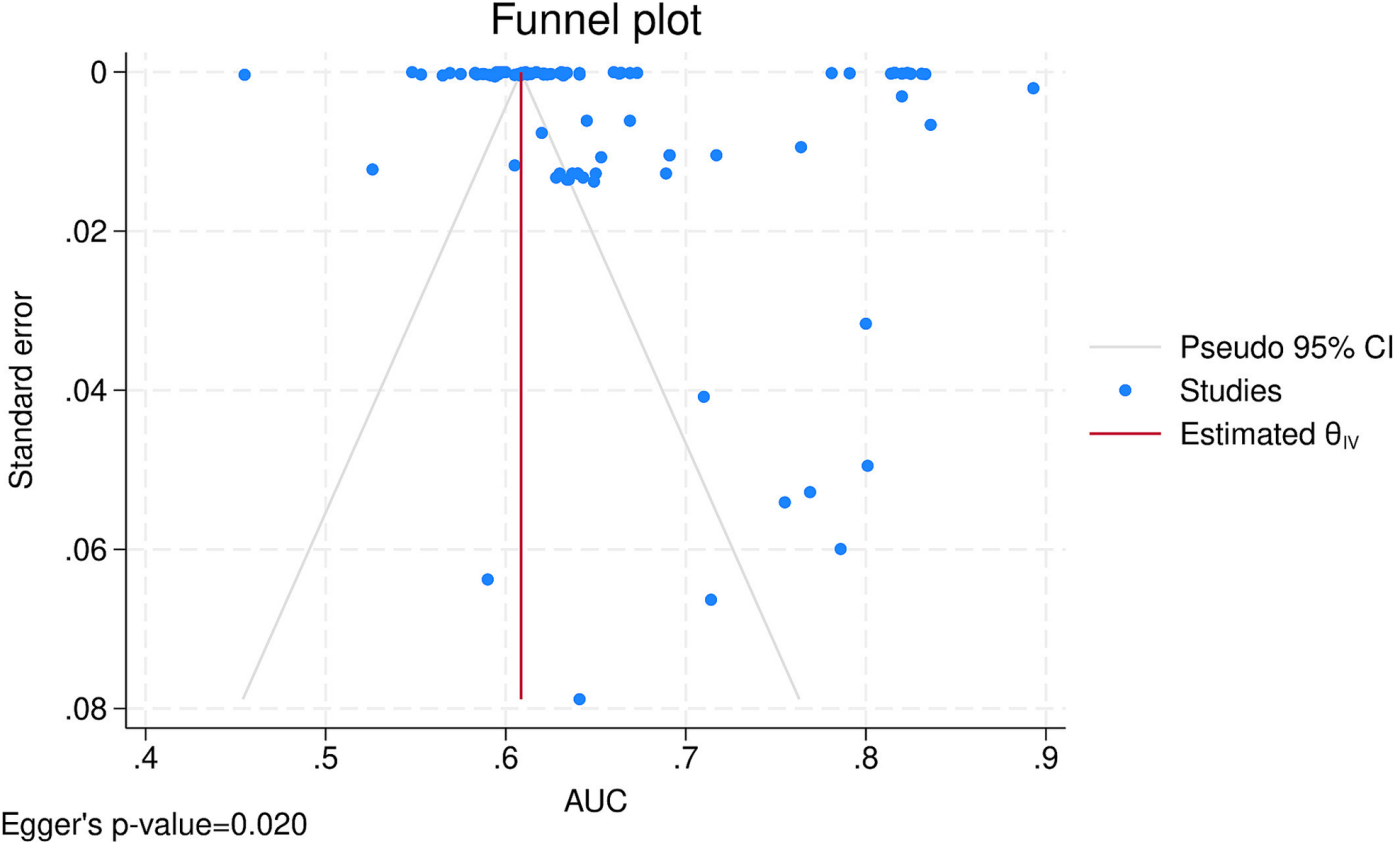
Funnel plot.

After combining the data from 12 (Nilsson et al., 2015; Dag et al., 2017; Medved et al., 2018; Yoon et al., 2018; Agasthi et al., 2020; Zhou et al., 2021; Kampaktsis et al., 2023; Kampaktsis et al., 2021; Ayers et al., 2021; Lisboa et al., 2022; Shou et al., 2022; Miller et al., 2022) studies in a meta-analysis, the overall AUC of all AI algorithms was 0.65 (95% CI: 0.64, 0.67), with externally validated models having an AUC of 0.64 (95% CI: 0.62, 0.65) and internally validated ones having an AUC of 0.65 (95% CI: 0.64, 0.67) and no significant subgroup difference (*p*-value = 0.10; Supplementary Figure S1). There was significant heterogeneity (I^2^ = 100.00%), which was investigated by a meta-regression of the time of mortality being predicted and subgroup analysis of the type of algorithm utilized. Meta-regression showed the longer the time since transplant is, the better the models perform (coefficient = 0.0005436, *p*-value = 0.008, R^2^ = 6.9%). Subgroup analysis also showed significant between-group differences for the type of algorithm (*p*-value<0.01) yet no difference between machine learning and deep learning algorithms (*p*-value = 0.67; Figure 2). Among the algorithms, K-nearest neighbors had the lowest AUC (0.53, 95% CI: 0.50, 0.55), whereas CatBoost had the highest AUC (0.80, 95% CI: 0.74, 0.86). Sensitivity analysis using the leave-one-out method indicates that our findings are stable (Supplementary Figure S2), yet publication bias was evident in the funnel plot (Figure 3; Egger’s *p*-value = 0.020). Further sensitivity analysis via exclusion of studies with a high risk of bias resulted in a similar pooled AUC of 0.62 (95% CI: 0.61, 0.64; data not shown).

**Figure 4:**
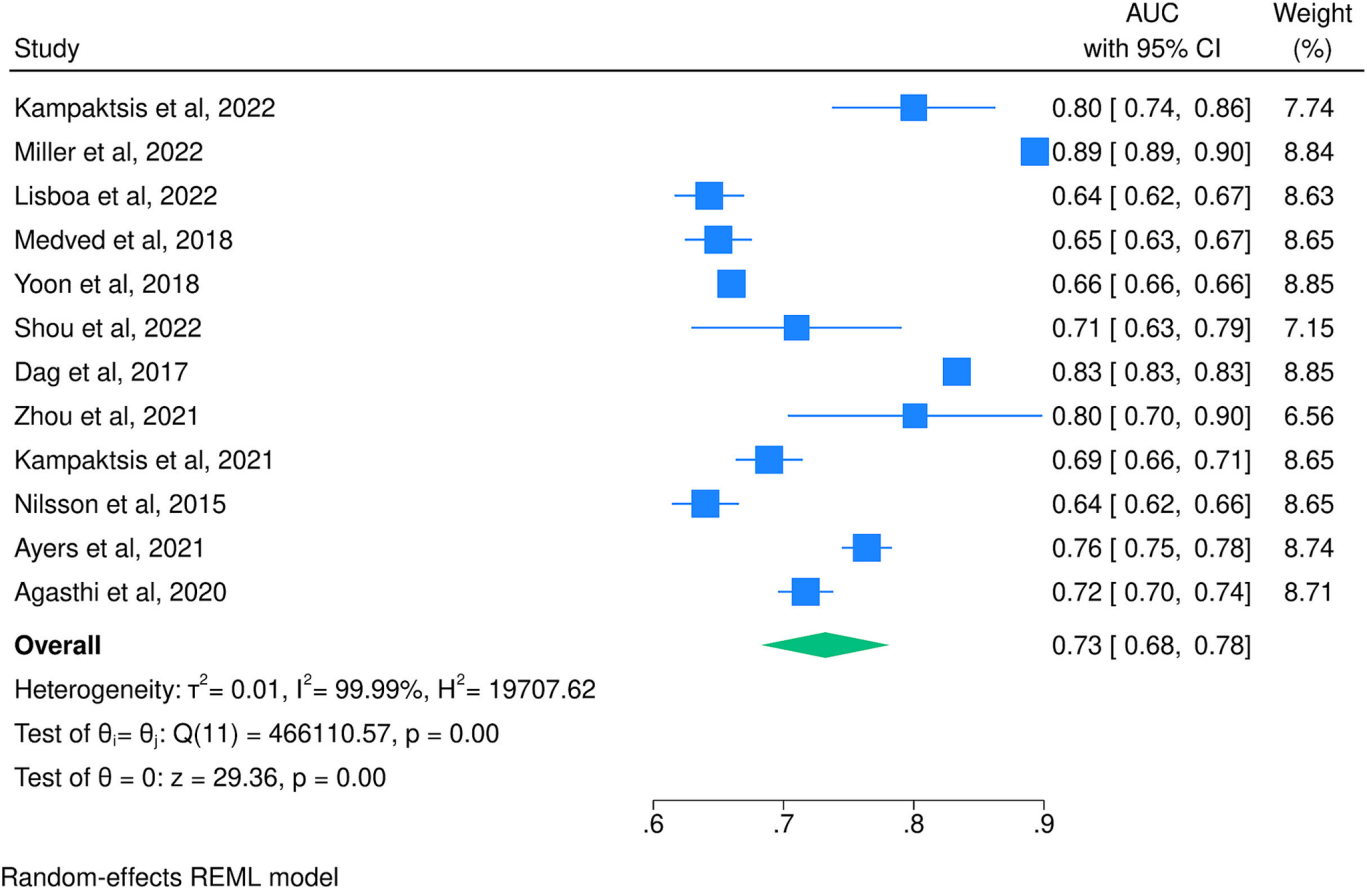
Meta-analysis of the area under the receiver operating characteristic curve for the best-performing models of each included study.

When pooling only the best-performing algorithms from each study, a pooled AUC of 0.73 (95% CI: 0.68, 0.78) was achieved with significant heterogeneity (I^2^ = 99.9%; Figure 4). From these, the most accurate model was developed by Miller et al. (2022), who used an RF model and achieved an AUC of 0.89 (95% CI: 0.89, 0.90), and the least accurate was developed by Nilsson et al. (2015), who used an ANN model and achieved an AUC of 0.64 (95% CI: 0.62, 0.66). Table 1 shows the detailed AUC values of the best-performing models from each of the 17 included studies, with models not included in the meta-analyses having AUC values ranging from 0.66 (Miller P. E. et al., 2019) to 0.95 (Raji and Safna, 2022). These five studies utilized RF (Miller R. et al., 2019; Killian et al., 2023; Ashfaq et al., 2023), NN (Miller P. E. et al., 2019), and ANN (Raji and Safna, 2022) and achieved slightly higher AUCs than those included in the meta-analyses.

### 3.4 Risk of bias assessment

**Figure 5:**
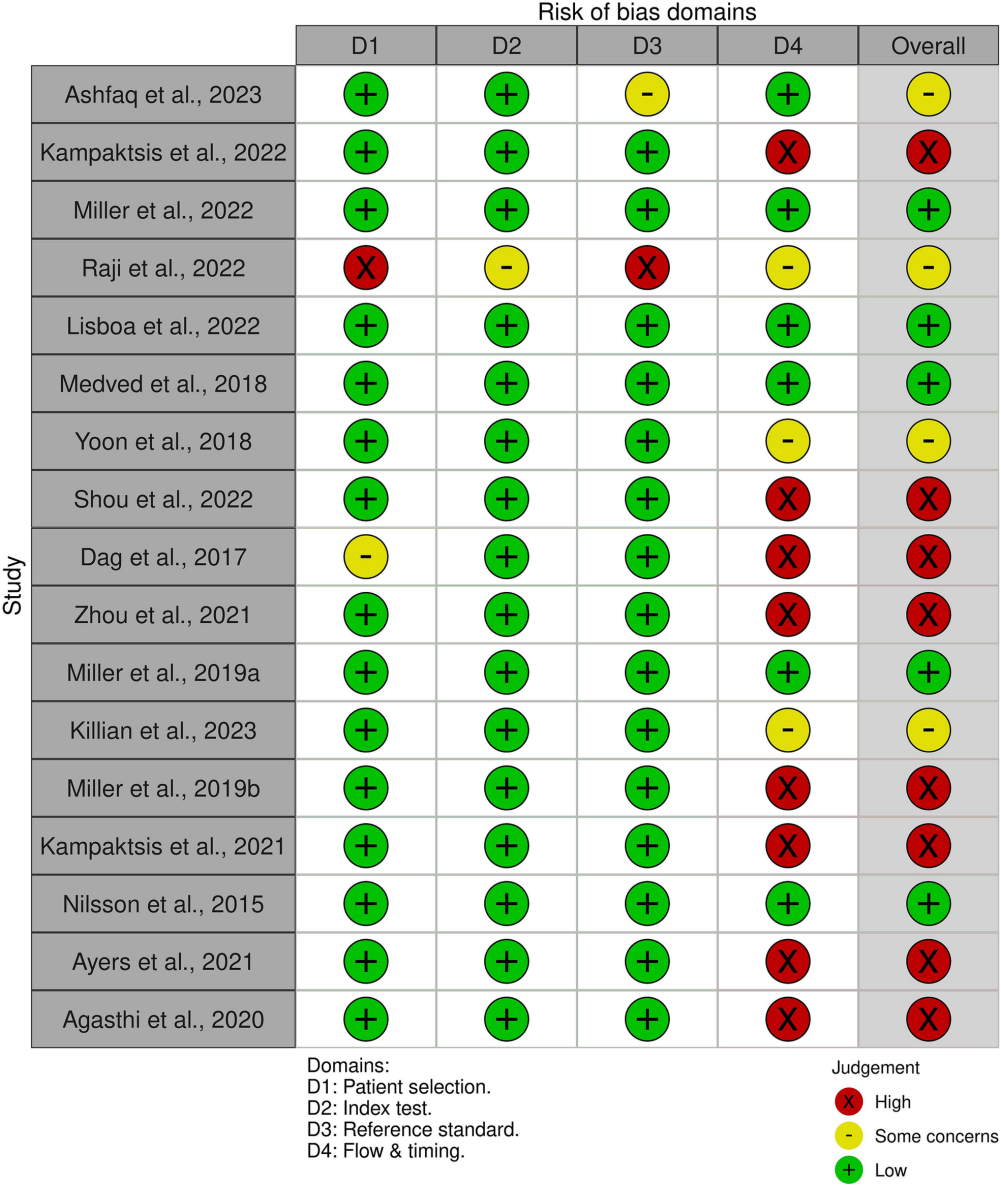
Risk of bias using the QUADAS-2 tool.

We utilized the QUADAS-2 tool to evaluate the risk of bias for all 17 studies in our review. Out of these studies, eight studies (Dag et al., 2017; Miller P. E. et al., 2019; Agasthi et al., 2020; Zhou et al., 2021; Kampaktsis et al., 2023; Kampaktsis et al., 2021; Ayers et al., 2021; Shou et al., 2022) were found to have a high risk of bias, while four (Yoon et al., 2018; Raji and Safna, 2022; Killian et al., 2023; Ashfaq et al., 2023) had an unclear risk of bias, primarily due to ambiguous analysis methods. The remaining five studies (Nilsson et al., 2015; Medved et al., 2018; Lisboa et al., 2022; Miller R. et al., 2019; Miller et al., 2022) were all assessed to be at a low risk of bias. Among the studies included in the meta-analysis, four had a low risk of bias (Nilsson et al., 2015; Medved et al., 2018; Lisboa et al., 2022; Miller et al., 2022), seven had a high risk of bias (Dag et al., 2017; Agasthi et al., 2020; Zhou et al., 2021; Kampaktsis et al., 2023; Kampaktsis et al., 2021; Ayers et al., 2021; Shou et al., 2022), and one study had an unclear risk of bias (Yoon et al., 2018). Figure 5 provides a summary of the risk of bias in the studies based on the four domains of QUADAS-2 tool. The most common cause of bias was in the flow and timing domains.

## 4 Discussion

Risk prediction is a crucial aspect of cardiovascular surgeries, especially in HT. Given the limited supply of donor organs, decisions about transplant eligibility and organ allocation are largely influenced by the predicted post-transplant risk of complications and mortality. Accurately assessing recipients’ mortality risk is crucial for optimizing organ allocation and ensuring the best donor-recipient matches. To this end, many models have been developed to predict mortality following HT in the literature. Although traditional regression-based models have been relatively successful in predicting mortality, ML models have shown great promise in surpassing them as they are better equipped to capture more complex non-linear interactions between characteristics. To better understand and compare these models, this systematic review and meta-analysis aimed to gauge the accuracy of ML models in predicting mortality following HT. We found that the overall predictive ability of the ML models was 0.65, and the meta-analysis of the best-performing algorithms from each study yielded a pooled AUC of 0.73. On average, CatBoost performed the best with an AUC of 0.80, whereas KNN performed the worst with an AUC of 0.53. Both traditional machine learning and deep learning algorithms performed similarly, and models performed better when a longer time had passed since the HT. Table 2 delineates the general advantages and disadvantages of the most widely used ML models in this study (Hornyák and Iantovics, 2023; Fort, 2018; Lantz, 2019; Sarker, 2021; Dangeti, 2017; Akinsola, 2017).

**Table 2:** The advantages and disadvantages of the most widely used models in our study.

ML method	Advantages	Disadvantages
Decision tree-based models	Handles complex data. Easy to interpret (DT). Robust to overfitting (RF).	Prone to overfitting (DT). Computationally expensive (RF). Less interpretable (RF).
Support vector machines	Effective in high-dimensional spaces. Works well with smaller datasets. Robust to overfitting with proper tuning.	Computationally slow with large datasets. Sensitive to kernel choice. Limited probabilistic output.
Adaptive boosting	Combines weak-learning algorithms to increase the accuracy Handles non-linear data well.	Sensitive to noisy data. Computationally demanding. Needs careful tuning.
Gradient boosting	Efficient and scalable. Handles missing data. Built-in regularization to prevent overfitting.	Requires careful hyperparameter tuning. Computationally expensive for large datasets.
Neural networks	Powerful in capturing deep patterns. Scalable for large datasets.	Requires significant computational resources. Hard to interpret and tune.

Although our pooled analysis revealed relatively low discrimination power among ML models, it is essential to contextualize their performance by comparing them with other established prediction models in the literature. The Donor Risk Index (DRI), the risk stratification score (RSS), and the Index for Mortality Prediction after Cardiac Transplantation (IMPACT) are three of the most prominent models that have been developed using logistic regression. Nilsson et al. compared the International Heart Transplantation Survival Algorithm (IHTSA) model to DRI, RSS, and IMPACT and found that IHTSA outperformed all three models in predicting 1-year mortality (Nilsson et al., 2015). Similarly, Medved et al. also found that the IHTSA showed superior discriminatory power to predict 1-year mortality and long-term survival after heart transplantation than the IMPACT (Medved et al., 2018). Additionally, an abstract by Yagi et al. externally validating both the IHTSA and the IMPACT found that the C-index for survival using the IMPACT score and 5-year mortality rate based on the IHTSA model were 0.689 and 0.720, respectively, denoting superiority of IHTSA (Yagi et al., 2020).

A range of variables were identified as significant contributors to mortality among the included studies, which can be grouped into categories such as recipient factors, donor factors, and transplant-related and post-operative factors.

Recipient characteristics, including functional status, age, specific diagnoses, and pediatric considerations, emerged as key predictors of mortality. Ashfaq et al. identified recipient functional status at listing as one of the most important predictors of 1-year mortality (Ashfaq et al., 2023). Similarly, Shou et al. reported that recipient functional status, age, and pulmonary capillary wedge pressure were the most predictive variables in their GBM model (Shou et al., 2022). Nilsson et al. highlighted recipient age and creatinine levels as critical predictors in the International Heart Transplantation Survival Algorithm (IHTSA) (Nilsson et al., 2015). Miller et al. also reported that bilirubin and creatinine levels at transplant were important predictors of mortality across LR, RF, and XGB models (Miller et al., 2022). Agasthi et al. and Lisboa et al. also found age to be an important recipient factor (Agasthi et al., 2020). Specific diagnoses also played an important role, as Miller et al.’s RF model found that congenital heart defect at listing was the most predictive variable for pediatric mortality at 1, 3, and 5 years. Additionally, cardiomyopathy and ECMO at transplant were predictive of 1-year mortality, with cardiomyopathy and bilirubin levels predictive of 3-year mortality (Miller R. et al., 2019). Kampaktsis et al.’s CatBoost model identified recipient age and eGFR as key predictors of 1-year mortality (Kampaktsis et al., 2023). Dag et al. emphasized the importance of recipient socioeconomic status, diagnosis for heart transplant at candidacy, and functional status at listing and transplant in predicting long-term mortality at 1, 5, and 9 years (Dag et al., 2017).

Donor characteristics were shown to significantly influence outcomes. Lisboa et al.’s partial response network–Lasso model identified donor age and ischemic time as highly predictive of 1-year mortality (Lisboa et al., 2022). Nilsson et al. similarly found donor age to be an important factor in their analysis (Nilsson et al., 2015). Miller et al.’s RF model additionally highlighted donor cytomegalovirus status and donor B1 antigen levels as predictors of 5-year mortality in pediatric patients (Miller R. et al., 2019).

Variables related to the transplant process, such as ventilator use, ischemic time, and graft status, were prominent in several models. Ashfaq et al. highlighted ventilator use at transplant as an important predictor of 1-year mortality (Ashfaq et al., 2023). Lisboa et al. and Agasthi et al. both identified ischemic time as a significant factor in 1- and 5-year mortality, respectively (Agasthi et al., 2020; Lisboa et al., 2022). Killian et al.’s RF model also found graft status and days in status 1A to be highly predictive of 1-, 3-, and 5-year mortality (Killian et al., 2023). Post-operative factors also contributed to the prognosis. Kampaktsis et al. emphasized post-operative hemodialysis as a top predictor of mortality in their CatBoost model (Kampaktsis et al., 2023). Agasthi et al. also identified hospital length of stay as a predictor (Agasthi et al., 2020).

Our study has several limitations. First, the cumulative AUC calculated (AUC = 0.65) implies that current AI models offer only a limited degree of clinical applicability, as it is generally agreed upon that in diagnostic value studies, AUC values above 0.90 indicate excellent performance, whereas AUC values below 0.80, even if statistically significant, imply a very limited clinical utility (White et al., 2023). Be that as it may, CatBoost has shown promise by achieving an AUC of 0.80, and future research is warranted to optimize this model. Second, a high degree of heterogeneity was observed when pooling the performance of the models. Our analysis was successful in attributing this heterogeneity to the type of the model and the time that has passed since the heart transplant. Other factors, such as population characteristics and type of disease, could have also contributed to this heterogeneity, as some of the studies used both adult and pediatric patients undergoing a range of procedures for their training. We could not perform subgroup analyses by population type, as the meta-analysis included only one pediatric study. Similarly, subgroup analysis by data source was unfeasible, as only one study in the meta-analysis did not rely on registries. In addition, feature selection, hyperparameter settings, and data preprocessing methods could have contributed to the heterogeneity, as a wide array of methods were used to construct the included models. For instance, in the case of feature selection, Ashfaq et al. (2023) used features that were selected by medical professionals, while Kampaktsis et al. (2021) used an ML feature selection method to do so. Furthermore, the models may have differed widely with respect to their hyperparameters, such as the number of trees in tree-based models, the number of layers and nodes used by NNs, or the number of cross-validation folds. In the case of data preprocessing, some simply excluded variables with too many missing values, whereas others used imputation to estimate the missing values without excluding them. Unfortunately, these aspects were not reported uniformly across different studies and, in some cases, were missing entirely from the reports. As a result, we were unable to explore them in subgroup analyses or meta-regressions. We suggest that future studies follow guidelines such as TRIPOD+AI (Collins et al., 2024) in order to enable future meta-analyses to assess the effect of these aspects of the models on their performance. Finally, most of the included studies were judged to be of low quality according to the QUADAS-2 tool. We recommend that future research be conducted in accordance with reporting and quality checklists in the literature to ensure the quality of analyses in future meta-analyses.

## 5 Conclusion

In conclusion, this systematic review and meta-analysis evaluated ML models for predicting mortality after heart transplantation (HT), yielding a pooled AUC of 0.73, with CatBoost performing best (AUC of 0.80). ML models demonstrated the potential to outperform traditional regression-based scores such as DRI, RSS, and IMPACT in capturing complex, non-linear interactions. However, high heterogeneity and variable study quality limit the reliability of pooled results. Key predictors of mortality include recipient diagnosis and functional status, age, and donor characteristics. Future studies should focus on improving methodological consistency and directly comparing ML approaches to traditional models to optimize clinical decision-making in HT.

## Data Availability

The original contributions presented in the study are included in the article/Supplementary material, further inquiries can be directed to the corresponding author.

## Supplementary material

SUPPLEMENTARY FIGURE S1 Forest plot of the area under the receiver operating characteristic curve of the included studies stratified by internal or external validation.

SUPPLEMENTARY FIGURE S2 Sensitivity analysis using the leave-one-out method.
