# Supplementary material for: Mortality prediction of heart transplantation using machine learning models: a systematic review and meta-analysis
Source: Front Artif Intell. 2025 Apr 4;8:1551959. doi: 10.3389/frai.2025.1551959 (PMC12006172; doi:10.3389/frai.2025.1551959)
Supplement: SUPPLEMENTARY FIGURE S1 — Forest plot of the area under the receiver operating characteristic curve of the included studies stratified by internal or external validation. [file Data_Sheet_1.pdf]

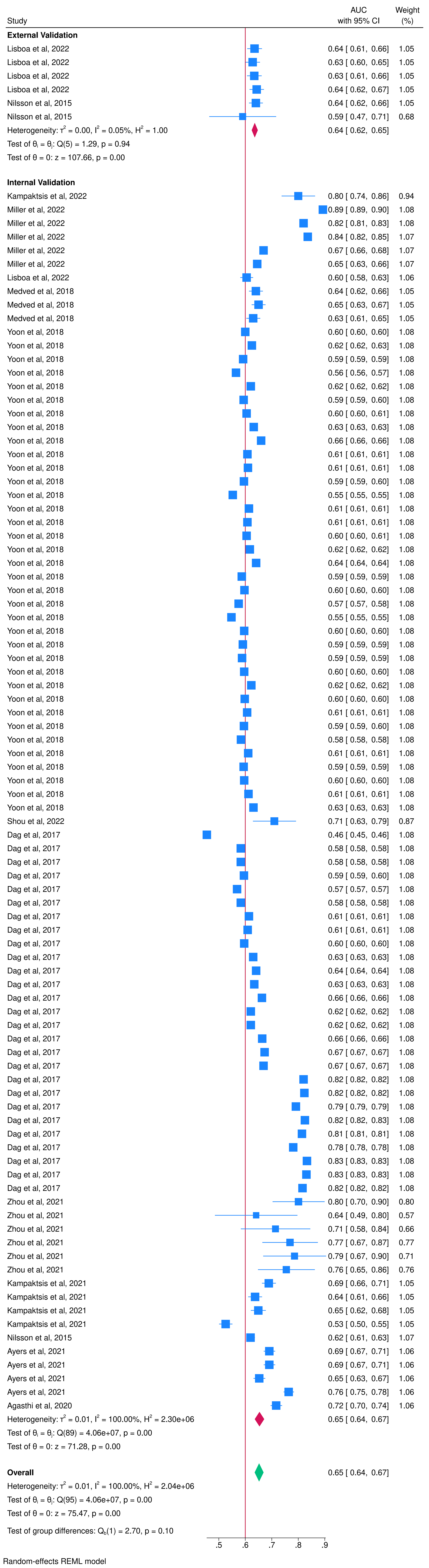
Study
AUC with 95% CI
Weight (%)
External Validation
Lisboa et al, 2022 0.64 [0.61, 0.66] 1.05
Lisboa et al, 2022 0.63 [0.60, 0.65] 1.05
Lisboa et al, 2022 0.63 [0.61, 0.66] 1.05
Lisboa et al, 2022 0.64 [0.62, 0.67] 1.05
Nilsson et al, 2015 0.64 [0.62, 0.66] 1.05
Nilsson et al, 2015 0.59 [0.47, 0.71] 0.68
Heterogeneity: τ^2 = 0.00, I^2 = 0.05%, H^2 = 1.00 0.64 [0.62, 0.65]
Test of $\theta_i = \theta_j$: Q(5) = 1.29, p = 0.94
Test of θ = 0: z = 107.66, p = 0.00
Internal Validation
Kampaktsis et al, 2022 0.80 [0.74, 0.86] 0.94
Miller et al, 2022 0.89 [0.89, 0.90] 1.08
Miller et al, 2022 0.82 [0.81, 0.83] 1.08
Miller et al, 2022 0.84 [0.82, 0.85] 1.07
Miller et al, 2022 0.67 [0.66, 0.68] 1.07
Miller et al, 2022 0.65 [0.63, 0.66] 1.07
Lisboa et al, 2022 0.60 [0.58, 0.63] 1.06
Medved et al, 2018 0.64 [0.62, 0.66] 1.05
Medved et al, 2018 0.65 [0.63, 0.67] 1.05
Medved et al, 2018 0.63 [0.61, 0.65] 1.05
Yoon et al, 2018 0.60 [0.60, 0.60] 1.08
Yoon et al, 2018 0.62 [0.62, 0.63] 1.08
Yoon et al, 2018 0.59 [0.59, 0.59] 1.08
Yoon et al, 2018 0.56 [0.56, 0.57] 1.08
Yoon et al, 2018 0.62 [0.62, 0.62] 1.08
Yoon et al, 2018 0.59 [0.59, 0.60] 1.08
Yoon et al, 2018 0.60 [0.60, 0.61] 1.08
Yoon et al, 2018 0.63 [0.63, 0.63] 1.08
Yoon et al, 2018 0.66 [0.66, 0.66] 1.08
Yoon et al, 2018 0.61 [0.61, 0.61] 1.08
Yoon et al, 2018 0.61 [0.61, 0.61] 1.08
Yoon et al, 2018 0.59 [0.59, 0.60] 1.08
Yoon et al, 2018 0.55 [0.55, 0.55] 1.08
Yoon et al, 2018 0.61 [0.61, 0.61] 1.08
Yoon et al, 2018 0.61 [0.61, 0.61] 1.08
Yoon et al, 2018 0.60 [0.60, 0.61] 1.08
Yoon et al, 2018 0.62 [0.62, 0.62] 1.08
Yoon et al, 2018 0.64 [0.64, 0.64] 1.08
Yoon et al, 2018 0.59 [0.59, 0.59] 1.08
Yoon et al, 2018 0.60 [0.60, 0.60] 1.08
Yoon et al, 2018 0.57 [0.57, 0.58] 1.08
Yoon et al, 2018 0.55 [0.55, 0.55] 1.08
Yoon et al, 2018 0.60 [0.60, 0.60] 1.08
Yoon et al, 2018 0.59 [0.59, 0.59] 1.08
Yoon et al, 2018 0.59 [0.59, 0.59] 1.08
Yoon et al, 2018 0.60 [0.60, 0.60] 1.08
Yoon et al, 2018 0.62 [0.62, 0.62] 1.08
Yoon et al, 2018 0.60 [0.60, 0.60] 1.08
Yoon et al, 2018 0.61 [0.61, 0.61] 1.08
Yoon et al, 2018 0.59 [0.59, 0.60] 1.08
Yoon et al, 2018 0.58 [0.58, 0.58] 1.08
Yoon et al, 2018 0.61 [0.61, 0.61] 1.08
Yoon et al, 2018 0.59 [0.59, 0.59] 1.08
Yoon et al, 2018 0.60 [0.60, 0.60] 1.08
Yoon et al, 2018 0.61 [0.61, 0.61] 1.08
Yoon et al, 2018 0.63 [0.63, 0.63] 1.08
Shou et al, 2022 0.71 [0.63, 0.79] 0.87
Dag et al, 2017 0.46 [0.45, 0.46] 1.08
Dag et al, 2017 0.58 [0.58, 0.58] 1.08
Dag et al, 2017 0.58 [0.58, 0.58] 1.08
Dag et al, 2017 0.59 [0.59, 0.60] 1.08
Dag et al, 2017 0.57 [0.57, 0.57] 1.08
Dag et al, 2017 0.58 [0.58, 0.58] 1.08
Dag et al, 2017 0.61 [0.61, 0.61] 1.08
Dag et al, 2017 0.61 [0.61, 0.61] 1.08
Dag et al, 2017 0.60 [0.60, 0.60] 1.08
Dag et al, 2017 0.63 [0.63, 0.63] 1.08
Dag et al, 2017 0.64 [0.64, 0.64] 1.08
Dag et al, 2017 0.63 [0.63, 0.63] 1.08
Dag et al, 2017 0.66 [0.66, 0.66] 1.08
Dag et al, 2017 0.62 [0.62, 0.62] 1.08
Dag et al, 2017 0.62 [0.62, 0.62] 1.08
Dag et al, 2017 0.66 [0.66, 0.66] 1.08
Dag et al, 2017 0.67 [0.67, 0.67] 1.08
Dag et al, 2017 0.67 [0.67, 0.67] 1.08
Dag et al, 2017 0.82 [0.82, 0.82] 1.08
Dag et al, 2017 0.82 [0.82, 0.82] 1.08
Dag et al, 2017 0.79 [0.79, 0.79] 1.08
Dag et al, 2017 0.82 [0.82, 0.83] 1.08
Dag et al, 2017 0.81 [0.81, 0.81] 1.08
Dag et al, 2017 0.78 [0.78, 0.78] 1.08
Dag et al, 2017 0.83 [0.83, 0.83] 1.08
Dag et al, 2017 0.83 [0.83, 0.83] 1.08
Dag et al, 2017 0.82 [0.82, 0.82] 1.08
Zhou et al, 2021 0.80 [0.70, 0.90] 0.80
Zhou et al, 2021 0.64 [0.49, 0.80] 0.57
Zhou et al, 2021 0.71 [0.58, 0.84] 0.66
Zhou et al, 2021 0.77 [0.67, 0.87] 0.77
Zhou et al, 2021 0.79 [0.67, 0.90] 0.71
Zhou et al, 2021 0.76 [0.65, 0.86] 0.76
Kampaktsis et al, 2021 0.69 [0.66, 0.71] 1.05
Kampaktsis et al, 2021 0.64 [0.61, 0.66] 1.05
Kampaktsis et al, 2021 0.65 [0.62, 0.68] 1.05
Kampaktsis et al, 2021 0.53 [0.50, 0.55] 1.05
Nilsson et al, 2015 0.62 [0.61, 0.63] 1.07
Ayers et al, 2021 0.69 [0.67, 0.71] 1.06
Ayers et al, 2021 0.69 [0.67, 0.71] 1.06
Ayers et al, 2021 0.65 [0.63, 0.67] 1.06
Ayers et al, 2021 0.76 [0.75, 0.78] 1.06
Agasthi et al, 2020 0.72 [0.70, 0.74] 1.06
Heterogeneity: τ^2 = 0.01, I^2 = 100.00%, H^2 = 2.30e+06 0.65 [0.64, 0.67]
Test of $\theta_i = \theta_j$: Q(89) = 4.06e+07, p = 0.00
Test of θ = 0: z = 71.28, p = 0.00
Overall 0.65 [0.64, 0.67]
Heterogeneity: τ^2 = 0.01, I^2 = 100.00%, H^2 = 2.04e+06
Test of $\theta_i = \theta_j$: Q(95) = 4.06e+07, p = 0.00
Test of θ = 0: z = 75.47, p = 0.00
Test of group differences: $Q_b(1)$ = 2.70, p = 0.10
.5 .6 .7 .8 .9
Random-effects REML model
