# Supplementary material for: Mortality prediction of heart transplantation using machine learning models: a systematic review and meta-analysis
Source: Front Artif Intell. 2025 Apr 4;8:1551959. doi: 10.3389/frai.2025.1551959 (PMC12006172; doi:10.3389/frai.2025.1551959)
Supplement: SUPPLEMENTARY FIGURE S2 — Sensitivity analysis using the leave-one-out method. [file Data_Sheet_2.pdf]

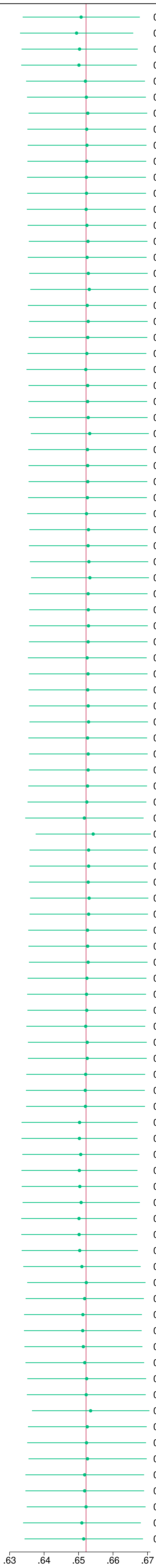

Omitted study	AUC with 95% CI	p-value
Kampaktsis et al, 2022	0.65 [0.63, 0.67]	0.000
Miller et al, 2022	0.65 [0.63, 0.67]	0.000
Miller et al, 2022	0.65 [0.63, 0.67]	0.000
Miller et al, 2022	0.65 [0.63, 0.67]	0.000
Miller et al, 2022	0.65 [0.63, 0.67]	0.000
Miller et al, 2022	0.65 [0.64, 0.67]	0.000
Lisboa et al, 2022	0.65 [0.64, 0.67]	0.000
Lisboa et al, 2022	0.65 [0.64, 0.67]	0.000
Lisboa et al, 2022	0.65 [0.64, 0.67]	0.000
Lisboa et al, 2022	0.65 [0.64, 0.67]	0.000
Lisboa et al, 2022	0.65 [0.64, 0.67]	0.000
Medved et al, 2018	0.65 [0.64, 0.67]	0.000
Medved et al, 2018	0.65 [0.64, 0.67]	0.000
Medved et al, 2018	0.65 [0.64, 0.67]	0.000
Yoon et al, 2018	0.65 [0.64, 0.67]	0.000
Yoon et al, 2018	0.65 [0.64, 0.67]	0.000
Yoon et al, 2018	0.65 [0.64, 0.67]	0.000
Yoon et al, 2018	0.65 [0.64, 0.67]	0.000
Yoon et al, 2018	0.65 [0.64, 0.67]	0.000
Yoon et al, 2018	0.65 [0.64, 0.67]	0.000
Yoon et al, 2018	0.65 [0.64, 0.67]	0.000
Yoon et al, 2018	0.65 [0.64, 0.67]	0.000
Yoon et al, 2018	0.65 [0.63, 0.67]	0.000
Yoon et al, 2018	0.65 [0.64, 0.67]	0.000
Yoon et al, 2018	0.65 [0.64, 0.67]	0.000
Yoon et al, 2018	0.65 [0.64, 0.67]	0.000
Yoon et al, 2018	0.65 [0.64, 0.67]	0.000
Yoon et al, 2018	0.65 [0.64, 0.67]	0.000
Yoon et al, 2018	0.65 [0.64, 0.67]	0.000
Yoon et al, 2018	0.65 [0.64, 0.67]	0.000
Yoon et al, 2018	0.65 [0.64, 0.67]	0.000
Yoon et al, 2018	0.65 [0.64, 0.67]	0.000
Yoon et al, 2018	0.65 [0.64, 0.67]	0.000
Yoon et al, 2018	0.65 [0.64, 0.67]	0.000
Yoon et al, 2018	0.65 [0.64, 0.67]	0.000
Yoon et al, 2018	0.65 [0.64, 0.67]	0.000
Yoon et al, 2018	0.65 [0.64, 0.67]	0.000
Yoon et al, 2018	0.65 [0.64, 0.67]	0.000
Yoon et al, 2018	0.65 [0.64, 0.67]	0.000
Yoon et al, 2018	0.65 [0.64, 0.67]	0.000
Yoon et al, 2018	0.65 [0.64, 0.67]	0.000
Yoon et al, 2018	0.65 [0.64, 0.67]	0.000
Yoon et al, 2018	0.65 [0.64, 0.67]	0.000
Yoon et al, 2018	0.65 [0.64, 0.67]	0.000
Yoon et al, 2018	0.65 [0.64, 0.67]	0.000
Yoon et al, 2018	0.65 [0.64, 0.67]	0.000
Yoon et al, 2018	0.65 [0.64, 0.67]	0.000
Yoon et al, 2018	0.65 [0.64, 0.67]	0.000
Yoon et al, 2018	0.65 [0.64, 0.67]	0.000
Yoon et al, 2018	0.65 [0.64, 0.67]	0.000
Shou et al, 2022	0.65 [0.63, 0.67]	0.000
Dag et al, 2017	0.65 [0.64, 0.67]	0.000
Dag et al, 2017	0.65 [0.64, 0.67]	0.000
Dag et al, 2017	0.65 [0.64, 0.67]	0.000
Dag et al, 2017	0.65 [0.64, 0.67]	0.000
Dag et al, 2017	0.65 [0.64, 0.67]	0.000
Dag et al, 2017	0.65 [0.64, 0.67]	0.000
Dag et al, 2017	0.65 [0.64, 0.67]	0.000
Dag et al, 2017	0.65 [0.64, 0.67]	0.000
Dag et al, 2017	0.65 [0.64, 0.67]	0.000
Dag et al, 2017	0.65 [0.64, 0.67]	0.000
Dag et al, 2017	0.65 [0.64, 0.67]	0.000
Dag et al, 2017	0.65 [0.64, 0.67]	0.000
Dag et al, 2017	0.65 [0.63, 0.67]	0.000
Dag et al, 2017	0.65 [0.64, 0.67]	0.000
Dag et al, 2017	0.65 [0.64, 0.67]	0.000
Dag et al, 2017	0.65 [0.63, 0.67]	0.000
Dag et al, 2017	0.65 [0.63, 0.67]	0.000
Dag et al, 2017	0.65 [0.63, 0.67]	0.000
Dag et al, 2017	0.65 [0.63, 0.67]	0.000
Dag et al, 2017	0.65 [0.63, 0.67]	0.000
Dag et al, 2017	0.65 [0.63, 0.67]	0.000
Dag et al, 2017	0.65 [0.63, 0.67]	0.000
Dag et al, 2017	0.65 [0.63, 0.67]	0.000
Dag et al, 2017	0.65 [0.63, 0.67]	0.000
Dag et al, 2017	0.65 [0.63, 0.67]	0.000
Dag et al, 2017	0.65 [0.63, 0.67]	0.000
Dag et al, 2017	0.65 [0.63, 0.67]	0.000
Zhou et al, 2021	0.65 [0.63, 0.67]	0.000
Zhou et al, 2021	0.65 [0.64, 0.67]	0.000
Zhou et al, 2021	0.65 [0.63, 0.67]	0.000
Zhou et al, 2021	0.65 [0.63, 0.67]	0.000
Zhou et al, 2021	0.65 [0.63, 0.67]	0.000
Zhou et al, 2021	0.65 [0.63, 0.67]	0.000
Kampaktsis et al, 2021	0.65 [0.63, 0.67]	0.000
Kampaktsis et al, 2021	0.65 [0.64, 0.67]	0.000
Kampaktsis et al, 2021	0.65 [0.64, 0.67]	0.000
Kampaktsis et al, 2021	0.65 [0.64, 0.67]	0.000
Nilsson et al, 2015	0.65 [0.64, 0.67]	0.000
Nilsson et al, 2015	0.65 [0.64, 0.67]	0.000
Nilsson et al, 2015	0.65 [0.64, 0.67]	0.000
Ayers et al, 2021	0.65 [0.63, 0.67]	0.000
Ayers et al, 2021	0.65 [0.63, 0.67]	0.000
Ayers et al, 2021	0.65 [0.64, 0.67]	0.000
Ayers et al, 2021	0.65 [0.63, 0.67]	0.000
Agasthi et al, 2020	0.65 [0.63, 0.67]	0.000

Random-effects REML model
